# New York Academy of Medicine

**Published:** 1860-03

**Authors:** 


### Selections.

---

### NEW YORK ACADEMY OF MEDICINE.

Condensed from Phonographic Reports for the Medical and Surgical Reporter.

### DISCUSSION ON ANÆSTHETICS.

Dr. Kissam related his experience with the two principal anæsthetics, sulphuric ether and chloroform, as employed in surgery; he gave preference to chloroform, but recommended it to be used with great caution.

Dr. Reese coincided with Dr. Kissam. As to the different action which the two articles possess, he would state that he was in the habit of using chloroform in puerperal convulsions, with good effect, while ether could not be used in this disease, because it rather increased, if any thing, the tendency to convulsions. In reference to chloroform, he had noticed that the Paris correspondent of the *New York Times*, who is a physician, had stated that the use of this remedy had been lately attended with exceedingly fatal results in the Parisian hospitals. It was Trousseau, he thought, who had introduced and sanctioned the administration of chloroform by an apparatus in which the inhalation is made through one nostril—the other nostril being left open for the reception of atmospheric air. Great stress is laid upon the fact, that chloroform, as thus administered through the open nostril, and in such a way as to admit the atmosphere, prevents any injurious results from the accumlative action of the remedy, which are so often alarming. He had never in his own hands had a case in which the chloroform was attended by a fatal result, or even approached to fatality. He always introduced the remedy very gradually, withdrawing it occasionally, and never carrying it so far as to produce profound stertor or coma, which in no case, where the anæsthetic was resorted to, he deemed it necessary to produce.

Dr. Squib had used chloroform for the past five or six years in many cases, perhaps as often as a hundred times, for various affections, and had never seen it followed by disastrous results. He is not in the habit of giving it very profusely, very often giving hardly enough to deprive the patient of sensibility or ordinary sensation. Used in this manner it has proved a safe agent in his hands. He had given it as long as 45—50 minutes at a time, without producing any dangerous effects. The danger from its use he considers to arise chiefly from the rash administration of it, being given in many cases so as to prevent the admission of air. When mixed with air it is certainly far more safe than when that due admixture of air is not given.

The President, Dr. Watson, stated that he would like to have the distinction pointed out between the results of the use of ether and of chloroform.

Dr. MINOR: During eight years experience in the administration of anæsthetics, in the hospital to which he was attached, the free admission of atmospheric air with the anæsthic agent has always been sucured, and he had never yet met with more than one case, which appeared in his opinion to be dangerous. In that instance there was suspended animation for five or ten minutes; the patient, however, finally recovered. As a general rule, the agent is administered according to the plan laid down by Dr. Metcalfe, some years ago, in a paper which was read before the Academy. In some cases Dr. M. had seen chloroform administered, by young gentlemen, rather in a careless manner; as they become interested in the operation, they are apt to apply the sponge too closely to the patient's nostrils, and thus prevent, to a great degree, the admixture of air with the vapor of the anæsthetic. He believed that where chloroform is given with due care, and the quantity regulated according to the peculiarities of the case, there will be comparatively little danger following its use; when, on the other hand, it is carelessly or ignorantly administered, it is a dangerous remedy, and is not unfrequently followed by fatal results; in fact, he believed that most of the fatal cases can be traced to a careless administration of the remedy. These remarks, of course, referred to chloroform alone. In many instances he had used a mixture of chloroform and ether, the per centage of the latter, however, being so small, that as far as the ultimate effects were concerned, it might be considered as chloroform.

Dr. PEASLEE remarked that in regard to the danger accompanying the administration of chloroform, a distinction must be made between its use in obstetric and in surgical practice. In the former, it is comparatively safe, because the paroxysm and labor pains react, to a certain extent, against the influence of the anæsthetic, while it is much more fatal in surgical practice. In regard to the two agents, he preferred ether; he had commenced with it, and used it up to the present time. In the first few trials, unpleasant effects were occasionally seen, which Dr. Peaslee now feels certain to have been due to the manner in which it was administered. His own experience coincided with the results of Dr. Snow's experiments, and convinced him that the safety of the administration of this agent depends *cæteris paribus*, upon the proper amount of atmospheric air being given with it. With this precaution, Dr. P. has never seen it followed by any unpleasant consequences whatever, and furthermore, he had never known, seen, read or heard of a case, where it was followed by unpleasant effects for more than a few hours. In some cases, he had given as much as two pounds of ether, and not unfrequently one pound. Chloroform he used with the utmost care, and took the precaution, mentioned by Dr. Snow, never to give it for more than twenty minutes at a time. In one case, however, even with these precautions, respiration was interrupted for three or four minutes, and for a time he thought that the patient was dead, and it was only by establishing artificial respiration that recovery finally took place.

Dr. O'Reily related four cases in which chloroform was administered, in all of which the most alarming symptoms took place suddenly, although the agent had been administered with care. By most energetic means, however, they were all restored.

Dr. Dalton made the following remarks:

Of course, Mr. President, I have very little experience with regard to the effects of these two agents upon the human subject, although I had the pleasure of witnessing the first operation in which ether was used as an anæsthetic agent. In my own practice, if you may call it such, the patients have been principally animals. I presume, however, that there is very little difference in their mode of operation on animals and on men. When I commenced, I, of course, used ether; but as ether requires to be given in a very large bulk, I soon found it very inconvenient, and commenced using chloroform in its stead, and found it very much more pleasant for myself, because it was more easily administered to the animals, and I continued to use it for a certain time. Very soon, however, I found that the animals would occasionally die, which I attributed to some imperfection in the mode of administering the agent. I continued the practice, but still the accident referred to would occasionally occur. Not to take up too much time in details, the simple fact is, that, at the end of six months, from the time I commenced its administration I abandoned it. Sometime afterward I again had occasion to use it; I gave it, but found that it was followed by the same results. Since that time I have given it up altogether, and instead of it I have used sulphuric ether. I think I may say, without exaggeration, that I am thoroughly convinced that there is a radical difference in the danger following the administration of these two substances. I am sure that chloroform is more dangerous to animals, at least; whether it is so in man or not, I do not know.

In order to understand this subject thoroughly, it is necessary that we should endeavor to ascertain the manner in which death results in the fatal cases. Death sometimes follows without any evident or traceable cause. It may occur from ether or chloroform by a very careless administration, or from an impurity of the article, provided that the patient breathes nothing but the vapor of the ether or the chloroform. Now in these cases, death is not attributable to the ether or chloroform; it is simply due to the want of atmospheric air. If you give a man a grain of opium and then stop his mouth and nostrils, he will of course die; but certainly not from the opium, but from the want of atmospheric air. The same is true of the administration of ether or chloroform. Therefore the first thing to be attended to, when we wish to prevent a fatal issue from the administration of these substances is to see that they are given mixed with a sufficient quantity of atmospheric air, and then one cause of death would be excluded.

Sometimes, however, even with all our precaution, we find the respiration and the heart stopping suddenly and the patient dead. It is an interesting question to know whether or not death is produced by the *stoppage of respiration* or *of the heart.* My own belief is, that in the case of chloroform, death is produced by paralysis of the heart. My reasons for this view are two-fold.

In the first place, if you moderately etherize or chloroformize an ainmal, carrying it carefully just up to the point of insensibility, and then open the walls of the chest as quickly as possible, the lungs of course will collapse and respiration be at an end, but the heart will continue to beat for a considerable length of time. If, on the other hand, you etherize or chloroformize an animal until respiration is stopped, and then open the chest, you will find the heart still beating, but very feebly. I have several times performed the following experiment, namely: to etherize an animal moderately, but enough to deprive it of all sensibility, then immediately the chest was opened and the animal laid aside; another animal was then etherized until death was produced, and on immediately opening the chest the heart was found still, while in the first animal it was yet beating. So far as this goes it tends to show, with a great deal of conclusiveness, that the fatal result is produced by a direct paralysis of the heart.

In experimenting thus with animals, I have had occasion to notice very frequently, when the anæsthetic is carried only to the stoppage of respiration, that the animals usually recover, and expect with confidence that respiration will begin again; but if, on noticing that the respiration is stopped, I find the heart itself still, I know that the animal is dead, although I have noticed, after the circulation is at an end, that it is sometimes re-established in a certain manner which is entirely characteristic, and being once seen, is very readily recognized. This is, however, entirely unavailing; the animal never recovers.

In my own experience, then, fatal results have followed both ether and chloroform. I have killed dogs and cats with ether and chloroform, but I am obliged to take a great deal of pains to produce this result with ether, whereas death often follows the use of chloroform, notwithstanding the best precautions. It has been said, that when death occurs from the administration of chloroform in the human subject, that it is attributable to giving it too rapidly or too abundantly; but while there are undoubtedly many cases in which injurious results follow from the non-admission of a sufficient amount of air, still I am of the opinion that the injurious or fatal results can not always be attributed to that cause, for the reason that these accidents have occurred in the practice of our best and most careful surgeons, who invariably exhibit this remedy with the utmost caution, and yet when every thing appears to be going on well, the patient suddenly dies. So far, we know of no precaution which will prevent the occasional occurrence of this accident.

Dr. O'Reily gave his theory of the fatal action of chloroform. In his opinion, the anæsthetic acted primarily upon the ganglionic nervous system, by which the proper process of endosmose and exosmose in the lungs is disturbed, and the air cells in the lungs are prevented from duly decarbonizing the blood.

Dr. Buck related the history of the death from chloroform which occured several years ago at the New York Hospital. The patient in that case was a perfectly healthy man. Two weeks before the unfortunate accident occurred, he had taken chloroform, which produced no disagreeable result whatever, neither were any greater precautions taken at this time than when the fatal issue occurred. In his case there was nothing whatever to forbid the use of this agent. Neither can the unfortunate result be attributed to the impurity of the chloroform, for the same article had been administered a day before to one of the patients with the very best effect. In this instance the quantity was by no means large, certainly less than a drachm. The accident occured very suddenly indeed. Dr. B. had barely time to order the assistant to bring a wet cloth, and then felt of his pulse, but it was gone. He did not know how any greater precautions could have been used than had been in this case.

Dr. Batchelder: Had tried experiments with chloroform upon himself frequently, by inhaling it through one nostril while the other was stopped, and then watching the effects closely. At first a singing in the head and ears were felt, then flashings of light before the eyes, assuming various shapes, were seen, and a prickling sensation was felt in the fingers. If carried beyond this point it begins to affect the voluntary muscles, so much so that in some instances he would have fallen down if he had not stopped inhaling the agent. In relation to the manner in which it produces death, he agreed entirely with Dr. Dalton's views. He thought that the heart did not become paralyzed until all the voluntary muscles become so. He had seen but two cases in which the agent produced alarming symptoms. They were both surgical, and were rescued by inducing artificial respiration.

Dr. Gardner regarded the mimimum dose of 20 drops, as generally stated in books, as too large. In one case he had seen a sufficient anæsthetic effect produced to allow the painless extraction of a tooth, by the inhalation of only five drops of chloroform.

Dr. McNulty observed that from the turn which the discussion had taken one would draw the inference that sulphuric ether was a perfectly harmless and safe agent, and could be administered almost *ad libitum.* He certainly had that impression very strongly. Last week, however, he had occasion to administer sulphuric ether to his own brother, and of course all possible precautions were used. He was put in the recumbent posture, and an abundance of atmospheric air was secured with the vapor inhaled. For the first five minutes the proportion of ether to air was certainly not more than 1 in 20, perhaps only 1 in 50. From that time its administration was carried on very gradually, and he was apparently coming under the influence of the anæsthetic very well indeed. Suddenly, when looking at his face, he was struck with its remarkable pallor. On feeling the pulse it was found a mere flutter ; the respiration had also ceased. The sponge was immediately taken away, and Marshall Hall's ready method resorted to. The patient gradually came to, but did not fully recover until six hours afterward. This case, the Doctor remarked, has certainly shaken my confidence in the safety and harmlessness of this remedy, very much indeed.

The President, Dr. Watson, inquired of Dr. Dalton whether he had found ether *fail* to produce all the effects that can be wished for from chloroform.

Dr. Dalton : It has never failed in my hands. My experience would lead me to believe that it is capable of producing all the effects of chloroform.

---

*Tests for the purity of Chloroform.*—M. Berthe gives the following directions, in the *Moniteur des Hospitaux :*—Chloroform may contain chloride of elaidine, alcohol, various chlorides, amylic and methylic combinations, and aldehyde. By adding caustic potash to chloroform containing chloride of elaidine, the compound is transformed into chloride of acetyle, the fœtor of which is immediately noticed. In order to ascertain the presence of all the other compounds which may be mixed with the chloroform, especially alcoholic compounds, pound a small quantity of bichromate of potash in a little chloroform, and add to this mixture a few drops of sulphuric acid. If the chloroform is pure, a reddish-brown precipitate of chromic acid is formed; if not pure, the acid is reduced, whilst the precipitate, or sometimes the liquid itself, assumes a green color, dependent on the presence of the sesquioxide of chrome.

